# A Rare Case of Renal Gastrinoma

**DOI:** 10.1100/tsw.2009.77

**Published:** 2009-06-30

**Authors:** Devendar Katkoori, Srinivas Samavedi, Merce Jorda, Norman L . Block, Murugesan Manoharan

**Affiliations:** ^1^ Department of Urology, Miller School of Medicine, University of Miami, FL, USA; ^2^ Department of Pathology, Miller School of Medicine, University of Miami, FL, USA

**Keywords:** gastrinoma, renal, extrapancreatic, ectopic

## Abstract

We present a rare case of renal gastrinoma. To the best of our knowledge, only one case of renal gastrinoma has been reported in the literature so far. An African American male was diagnosed with Zollinger Ellison syndrome at the age of 15 years, when he underwent surgery for peritonitis secondary to duodenal ulcer perforation. Further evaluation was deferred and proton pump inhibitors were prescribed. Later evaluation showed a left renal mass. Serum gastrin levels were 4,307 pg/ml. A CAT scan of the abdomen showed 4- x 4-cm heterogeneous solid mass in the interpolar region of the left kidney with central hypodensity. Somatostatin scintigraphy confirmed a receptor-positive mass in the same location. Nephrectomy was done and the tumor was diagnosed on histopathological examination as a gastrinoma. At 6-month follow-up, gastrin levels were 72 pg/ml. After a follow-up of 6 years, the patient has no recurrent symptoms.

## INTRODUCTION

Since 1955, more than 1,000 patients with Zollinger Ellison syndrome (ZES) have been reported, with an estimated annual incidence of one per million population[1]. ZES is usually diagnosed in the fifth decade and is rare in the pediatric age group. The most common location for a gastrinoma is the duodenum. Ectopic gastrinomas have been reported in the heart, stomach, ovary, liver, kidney, and bile duct[1]. ZES secondary to gastrinoma of the kidney is extremely rare. It occurs in younger age group and, so far, only one case has been reported[2]. In the previously reported case, symptoms were secondary to hypersecretion of gastrin, leading to intractable duodenal ulcerations. Diagnosis was made by elevated serum gastrin levels, elevated selective renal vein gastrin assays, and a renal mass on CAT scan. Nephrectomy reduced serum gastrin levels; however, it did not reduce the basal acid output from the stomach[2].

## CASE REPORT

A 15-year-old, morbidly obese, African American male presented with abdominal pain in 1997. Physical examination showed signs of peritonitis. A laparotomy was done and a perforated duodenal ulcer was diagnosed; an omental patch repair was done. He underwent a secretin stimulation test, which was positive with high gastrin levels following intravenous secretin at 5, 10, and 15 min post-test. The maximum gastrin level postsecretin was more than 1,000 pg/ml, consistent with a diagnosis of ZES. He had no family history of ulcer disease. Evaluation for surgical management of ZES was deferred; proton pump inhibitors (omeprazole 20 mg thrice daily, later increased to 60 mg twice daily) and oral sucralfate (1 g every 6 h) were initiated. However, he presented 1 year later with recurrent hemetemesis and an upper gastrointestinal endoscopy was done. Prominent gastric folds, moderate to severe distal esophagitis with linear erosions, exudates, classified as grade III, were seen. Computerized tomography (CT) of the abdomen showed a 3-cm renal mass lesion on the left side. A CT-guided biopsy of the mass was done on two occasions and no malignancy was found. The renal mass was monitored by serial imaging. In February 2002, his gastrin levels were 4,307 pg/ml (normal gastrin levels: fasting <100 pg/ml, nonfasting <200 pg/ml) and attempts to localize the gastrinoma previously with a CT scan of the abdomen and endoscopic ultrasound were unsuccessful. A CT scan (Fig. 1A) showed increase in the size of the renal tumor to 4 cm, with a central hypodense area. Somatostatin scintigraphy was done and a somatostatin receptor-positive mass lesion was seen in the same region as on the CT scan (Fig. 1B). A left nephrectomy was done. Histopathology was consistent with a diagnosis of gastrinoma. The tumor cells were positive for chromogranin A, synaptophysin, and gastrin (Fig. 2), and were negative for pancreatic polypeptide (PPP) and gastrin-releasing peptide (GRP) by histochemistry. Capsular and vascular invasion was noted. Postoperatively, at 6-month follow-up, serum gastrin levels decreased to 72 pg/ml. After a follow-up of 6 years, the patient has no recurrent symptoms.

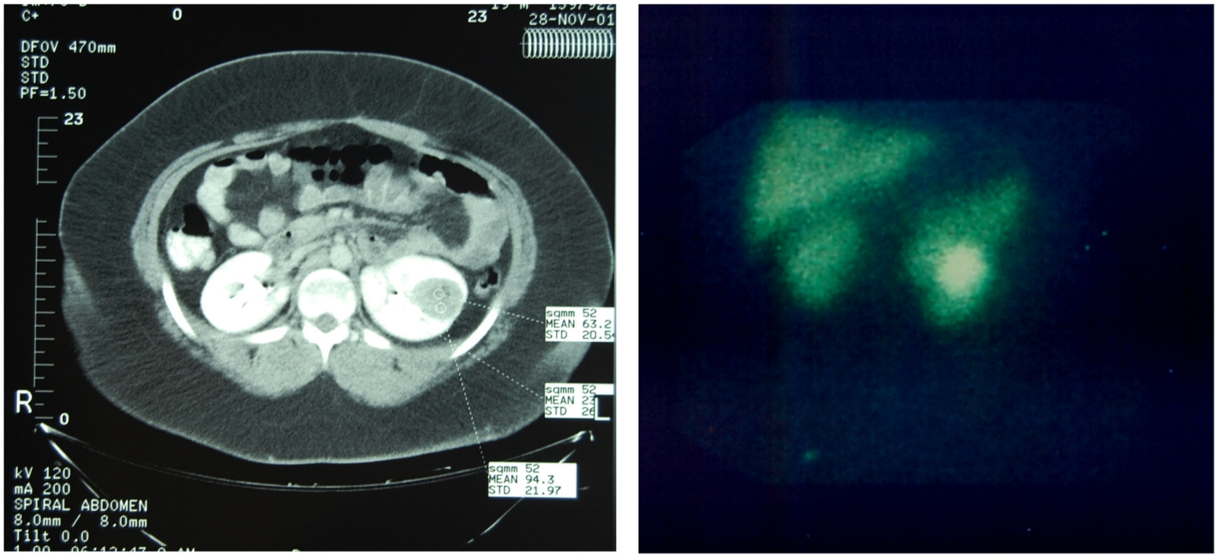

A B

**FIGURE 1**. (A) CT scan of the abdomen showing a 4- x 4-cm renal mass lesion in the interpolar region of the left kidney with central hypodensity. (B) Indium-111–labeled octreotide scan showing a focus of increased uptake in the mid-portion of left kidney consistent with a somatostatin receptor-positive mass.

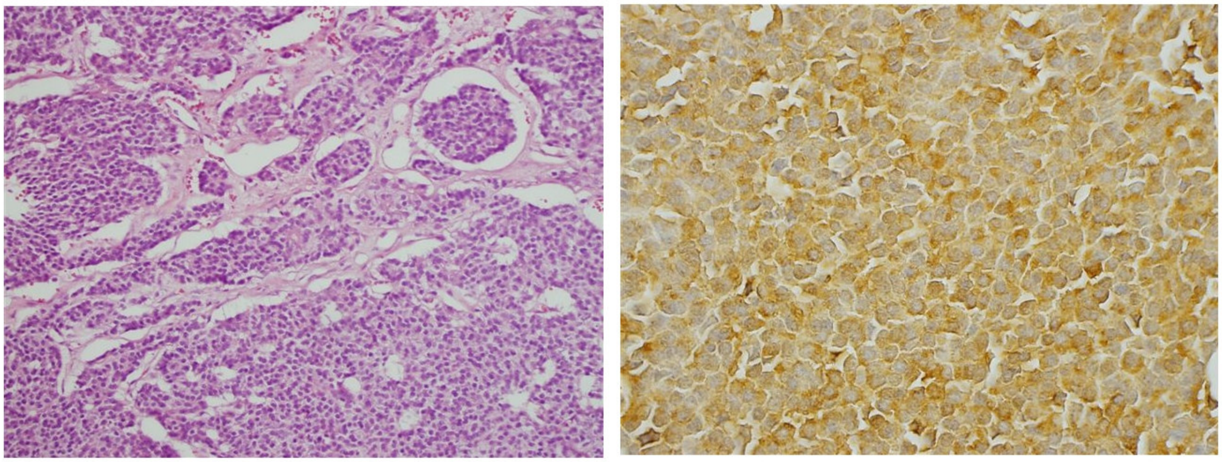

A B

**FIGURE 2.** (A) Hematoxylin & Eosin stain, 25×. (B) Tumor tissue stain positive for gastrin, 60×.

## DISCUSSION

This case is one of the two reported in the literature. The previously reported case of renal gastrinoma was a 12-year-old male, with a renal mass on imaging. The diagnosis was made by selective renal vein gastrin assay. In our case, a renal vein assay was not done due to a lack of suspicion and this may have been a potential cause for delay in treatment. Also, CT-guided biopsy had been inconclusive in two instances, emphasizing the need for selective renal vein gastrin assays. The paucity of staining for PPP was as described in the literature for ectopic extrapancreatic gastrinomas.

The totipotentiality of the fetal stem cell that persists into adulthood is thought to be responsible for gastrinomas arising in the kidney. Sporadic gastrinomas are found predominantly within the "*gastrinoma triangle*", defined as the confluence of cystic and common bile duct superiorly, the second and third portions of the duodenum inferiorly, and the neck and body of the pancreas medially, both dorsally and ventrally. It is postulated that sporadic gastrinomas in the triangle arise from stem cells of the ventral pancreatic bud that become dispersed, and incorporated into lymph tissue and the duodenal wall during the ventral bud's embryonic dorsal rotation within the area of the triangle. A recent report suggested a different genetic background for duodenal vs. pancreatic gastrinomas[3]. Ectopic gastrinomas outside the gastrinoma triangle are rare and would be found predominantly on the right side of the abdomen[4]. Eleven cases of ectopic extrapancreatic gastrinomas described in the literature were situated in the right-side ovary. Ovarian tissue is considered to be pluripotential and, in fact, has been associated with many endocrine tumors, such as thyroid or adrenal gland tumors. Gastrinomas found outside the gastrinoma triangle, which presumably have a different origin than those derived from the ventral pancreatic bud, may have different clinical characteristics or biological behavior. Tumors thought to be of ventral pancreatic bud origin are commonly found within lymph nodes, often extrapancreatic in location, and only rarely metastasizing to the liver, whereas tumors found in the body and tail of the pancreas (dorsal bud) are rarely within lymph nodes and are never found in extrapancreatic locations. Metastases to the liver are frequent with sporadic gastrinomas found outside the gastrinoma triangle (nonventral pancreatic bud origin), as is death from the tumor; both supporting the notion that these gastrinomas behave differently from those found within the gastrinoma triangle[5]. An important aspect of this theory is that a fetal cell present in the adult is being implicated in tumor (gastrinoma) formation. This requires that such fetal cells persist in adults[6].

The discovery of renal gastrinoma is a life-saving diagnosis for the patient. This diagnosis should be considered in all patients with high gastrin levels and a solid mass in the kidney. Because of the lack of typical radiographic and cytological findings, the correct preoperative diagnosis is impossible in most cases and a high degree of suspicion is necessary. Somatostatin receptor scintigraphy is a sensitive method for detection and is an important tool when a gastrinoma is suspected. A selective renal vein gastrin assay may be used to establish the diagnosis in difficult cases. Nephrectomy is usually curative.

## ACKNOWLEDGMENTS

Support was received from “CURED” and Mr. Vincent A. Rodriguez.

## References

1. Ellison, E.C. and Johnson, J.A. (2009) The Zollinger-Ellison syndrome: a comprehensive review of historical, scientific, and clinical considerations. *Curr. Probl. Surg.* **46,** 13–106.
2. Nord, K.S., Joshi, V., Hanna, M., Khademi, M., Saad, S., Marquis, J., Pelzman, H., and Verner, E. (1986) Zollinger-Ellison syndrome associated with a renal gastrinoma in a child. *J. Pediatr. Gastroenterol. Nutr.* **5,** 980–986.
3. Fendrich, V., Ramerth, R., Waldmann, J., Maschuw, K., Langer, P., Bartsch, D.K., Slater, E.P., Ramaswamy, A., and Rothmund, M. (2009) Sonic hedgehog and pancreatic-duodenal homeobox 1 expression distinguish between duodenal and pancreatic gastrinomas. *Endocr. Relat. Cancer* **16,** 613–622.
4. Sawicki, M.P., Howard, T.J., Dalton, M., Stabile, B.E., and Passaro, E., Jr. (1990) The dichotomous distribution of gastrinomas. *Arch. Surg*. **125,** 1584–1587.
5. Howard, T.J., Sawicki, M., Lewin, K.J., Steel, B., Bhagavan, B.S., Cummings, O.W., and Passaro, E., Jr. (1995) Pancreatic polypeptide immunoreactivity in sporadic gastrinoma: relationship to intraabdominal location. *Pancreas* **11,** 350–356.
6. Passaro, E., Jr., Howard, T.J., Sawicki, M.P., Watt, P.C., and Stabile, B.E. (1998) The origin of sporadic gastrinomas within the gastrinoma triangle: a theory. *Arch. Surg.* **133,** 13–16; discussion 17.
